# Secondary Vesical Calculus Resulting from Migration of an Intrauterine Contraceptive Device

**DOI:** 10.1155/2012/603193

**Published:** 2012-07-18

**Authors:** Suvarna Vagholkar, Ketan Vagholkar

**Affiliations:** Department of Surgery, Dr. Vagholkar Hospital, Annapurna Niwas, 229 Ghantali Road, Thane 400602, India

## Abstract

Intrauterine contraceptive device (IUCD) is the commonest form of contraception used in view of less systemic side effects. However, there are a multitude of local complications caused by it. Of all the local complications described, migration of the device into adjacent organs is the most morbid of all complications. A patient presenting with history of loss or disappearance of the intrauterine contraceptive device accompanied by urinary symptoms should raise the doubt of a migrated device with the formation of a secondary calculus. This prompts further radiological investigations and merits surgical intervention either endourologically or by open surgery depending upon the merits of the case. A case report elucidating this fact is presented.

## 1. Introduction

Intrauterine contraceptive devices are still commonly used. Copper T is the commonest intrauterine device used in India. Though the contraceptive effect of the device is great, yet the complications rate may at times outweigh the beneficial effects on contraception prevention. A multitude of complications are caused by these devices. However, migration of the device into adjacent organs is the most morbid of all complications. 

A case of transmigration of an intrauterine contraceptive device (copper T) into the bladder with formation of a secondary calculus around it is presented along with a review of the literature.

## 2. Case Report

A 50-year-old female patient presented with complaints of urinary frequency and dysuria. She also complained of occasional episodes of suprapubic pain. There was no history of hematuria, graveluria, or fever. Past history of the patient revealed that she had undergone dilatation and curettage after her last pregnancy at her native place followed by insertion of an intrauterine copper T device. This was 18 years back. She had no symptoms related to the reproductive system thereafter. The patient had completely forgotten about the intrauterine device since insertion. 

**Figure 1:**
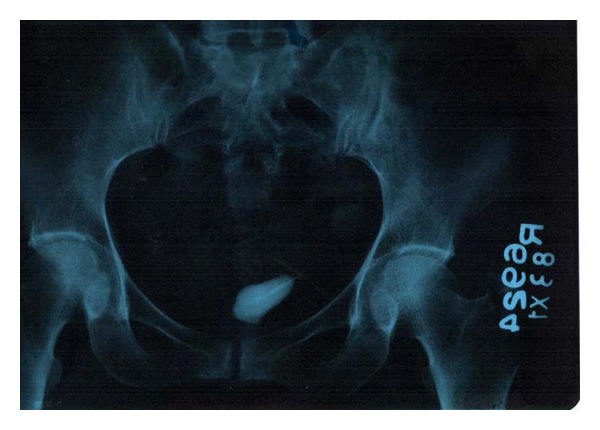
Plain X-ray showing the calculus and the horizontal limb of the device attached to it.

**Figure 2:**
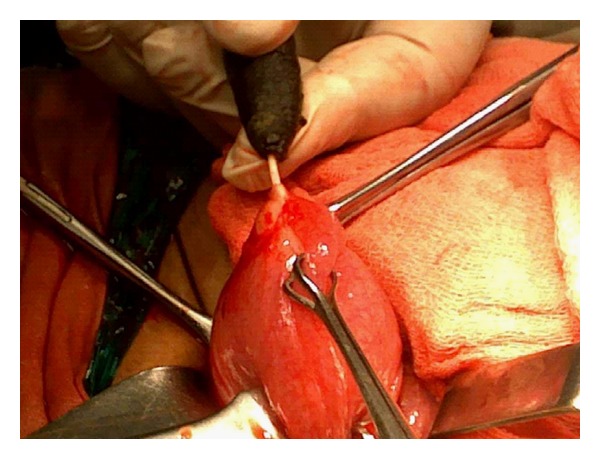
The stone was partially impregnated into the bladder musculature by virtue of the horizontal limb of the device.

**Figure 3:**
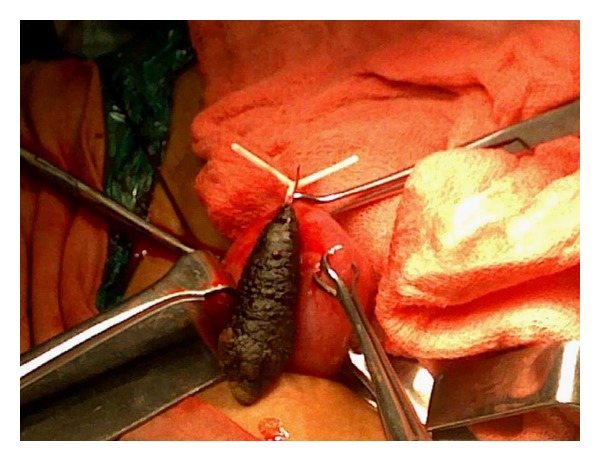
Complete removal of the stone along with the projecting horizontal limb of the intrauterine device.

Abdominal examination revealed tenderness on deep palpation over the suprapubic region. Per vaginal examination was normal and did not reveal any abnormality. Per rectal digital and proctoscopic examination did not reveal any abnormality. Hematological investigations were within the normal limits. Urine examination revealed abundant pus cells and microscopic hematuria. Plain X-ray revealed a calculus in the region of the bladder, measuring approximately 3 cms in length and 1.5 cms in breadth. At one end of the calculus, the horizontal limb of the device was seen connected to the vertical limb around which the calculus had formed (Figure 1). USG examination confirmed the presence of the calculus in the bladder which was fixed as it did not exhibit any mobility within the bladder. The patient was given a course of urinary antibiotics to control the infection and then subjected to surgery. A preliminary cystoscopic examination was performed. It was observed that the stone was firmly fixed to the postero superior wall of the bladder. In view of the fixity of the stone, an open suprapubic cystolithotomy was performed. The stone was partially impregnated into the bladder musculature by virtue of the horizontal limb of the device (Figure 2). The stone along with the projecting horizontal limb was carefully removed (Figure 3). The partial breach in bladder musculature caused by the impalement of the foreign body (device) was sutured with an absorbable suture material. The bladder was closed with absorbable suture material. Postoperative recovery was uneventful.

## 3. Discussion

Intrauterine contraceptive device (IUCD) has been used as a safe, successful, and economical method of contraception. There have been many studies which reveal significant number of complications such as bleeding irregularities, infection, perforation, and migration into adjacent organs [1, 2]. There are many reports stating the migration of the IUCD into the urinary bladder [3–6]. The uterus is normally in an anteverted and anteflexed position. By virtue of this position it lies in close proximity to the urinary bladder rather than the rectum. Hence, there is a high propensity of migration to the bladder. The process of transmigration of foreign bodies across organs is quite complex and at times difficult to understand. What is important for the surgeon to know is that no organ is impervious to foreign body migration. Once in the bladder, the symptoms pertaining to the urinary system will be experienced, such as frequency, dysuria, hematuria, retention of urine, and fever due to infection. If severe and longstanding it can cause serious back pressure changes. If the foreign body remains for a long time in the bladder, a secondary calculus is formed as seen in the reported case. Other causes of secondary stone formation reported are due to stents forgotten to be removed, pieces of teflon sheaths of resectoscope, and remnants of a ruptured Foley's catheter balloon or tip [2]. The symptoms are usually underestimated, and the patient is treated for urinary tract infection without any imaging investigation being carried out. Only when symptoms assume serious proportions, the patient undergoes further investigations. Hence, it would be a good practice to carry out a simple investigation like a plain X-ray in patients who have undergone any such pelvic procedure presenting with urinary symptoms. An X-ray and USG examination of the pelvis are enough to arrive to the diagnosis. If on USG the foreign body is found to involve more than one organ at the same time then CT scan should be done to study the exact site, size, and the pathway of the fistulous track caused by the migrating foreign body in order to plan optimum treatment [7].

 Laboratory investigations will reveal leukocytosis in infected cases, while urine examination will reveal either pus cells or microscopic or gross hematuria depending upon the severity of infection and the irritation caused by it. Surgery is the mainstay of treatment [7, 8]. It is a good and safe practice to administer a course of antibiotics prior to surgical intervention. A preliminary cystoscopic examination is mandatory to chart the course of further intervention [8]. If the stone around the foreign body is mobile then endoscopic methods for removal will suffice. However, if the stone is large and fixed then open suprapubic cystolithotomy is indicated. This allows safe and complete removal of the stone and any projecting bits of the foreign body as was done in the present case. It also allows for a good approximation of the defect caused by the impregnating foreign body and stone. 

In conclusion, a patient with either a lost IUCD or in whom urological intervention has been performed presenting with urinary symptoms should raise the suspicion of a secondary calculus around a retained foreign body necessitating further radiological investigation for confirmation followed by surgical intervention.
